# Lysozyme–Sucrose Interactions in the Solid State: Glass Transition, Denaturation, and the Effect of Residual Water

**DOI:** 10.1021/acs.molpharmaceut.3c00403

**Published:** 2023-08-09

**Authors:** Ekaterina Bogdanova, Sebastian Lages, Tuan Phan-Xuan, Md. Arif Kamal, Ann Terry, Anna Millqvist Fureby, Vitaly Kocherbitov

**Affiliations:** † Biomedical Science, Malmö University, Malmo SE-20506, Sweden; ‡ Biofilms research center for Biointerfaces, Malmo SE-20506, Sweden; § RISE Research Institutes of Sweden, Stockholm SE-114 86, Sweden; ∥ MAX IV Laboratory, Lund University, Lund SE-22484, Sweden; ⊥ Division of Physical Chemistry, Lund University, Box 124, Lund SE-221 00, Sweden

**Keywords:** solid-state formulations, proteins, hydration, glass transition, small-angle X-ray scattering, differential scanning calorimetry

## Abstract

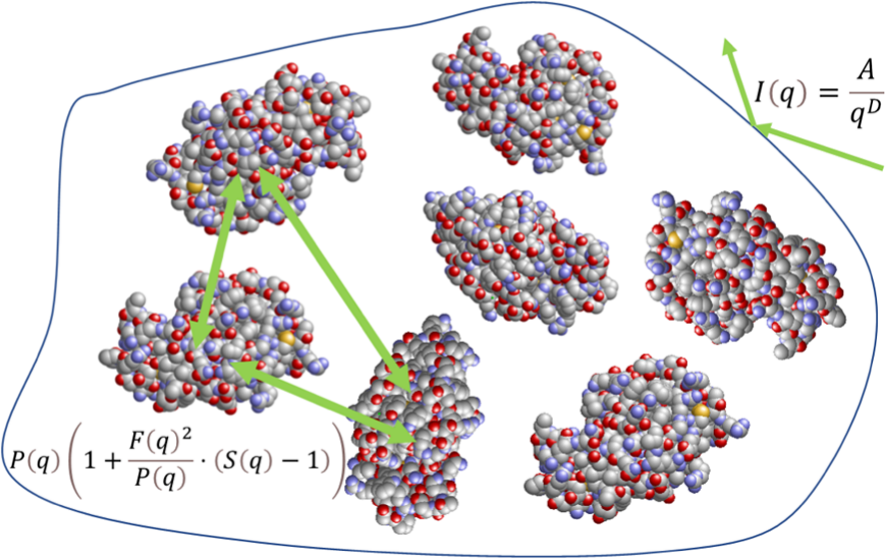

The freeze-drying of proteins, along with excipients, offers a solution for increasing the shelf-life of protein pharmaceuticals. Using differential scanning calorimetry, thermogravimetric analysis, sorption calorimetry, and synchrotron small-angle X-ray scattering (SAXS), we have characterized the properties at low (re)hydration levels of the protein lysozyme, which was freeze-dried together with the excipient sucrose. We observe that the residual moisture content in these samples increases with the addition of lysozyme. This results from an increase in equilibrium water content with lysozyme concentration at constant water activity. Furthermore, we also observed an increase in the glass transition temperature (*T*_g_) of the mixtures with increasing lysozyme concentration. Analysis of the heat capacity step of the mixtures indicates that lysozyme does not participate in the glass transition of the sucrose matrix; as a result, the observed increase in the *T*_g_ of the mixtures is the consequence of the confinement of the amorphous sucrose domains in the interstitial space between the lysozyme molecules. Sorption calorimetry experiments demonstrate that the hydration behavior of this formulation is similar to that of the pure amorphous sucrose, while the presence of lysozyme only shifts the sucrose transitions. SAXS analysis of amorphous lysozyme–sucrose mixtures and unfolding of lysozyme in this environment show that prior to unfolding, the size and shape of lysozyme in a solid sucrose matrix are consistent with its native state in an aqueous solution. The results obtained from our study will provide a better understanding of the low hydration behavior of protein–excipient mixtures and support the improved formulation of biologics.

## Introduction

Protein-based formulations are used more and more frequently in medical care. Some proteins do not exhibit satisfactory stability in liquid formulations and therefore they are dried to solid-dosage forms. To prevent proteins from losing their activity upon drying and storage, excipients are needed. It was shown that disaccharides such as sucrose and trehalose act as stabilizers for many proteins. Unfortunately, the mechanisms by which sugars stabilize proteins in the solid state have not been completely understood yet. Over the past few decades, two main theories have been put forward in the literature that attempts to describe the mechanisms of protein stabilizations by sugars in solid-state.^1−4^ Water replacement theory accounts for intermolecular interactions. In an aqueous solution, the native state of a protein is stabilized by hydrogen bonds between the protein and water molecules. Upon dehydration, these water molecules are replaced by sugar molecules, which form a hydrogen-bonded network quite similar to the water network around the protein. The vitrification or glass dynamics theory, on the other hand, is based on the physical immobilization of the protein inside a glassy sugar matrix, inhibiting protein movements and thereby leading to a dramatic increase in the denaturation time.

Disaccharides are known to affect the structural and colloidal stability of lysozyme in aqueous liquid solutions.^5,6^ For liquid solutions, when both sucrose and water are present in large amounts and compete for the interactions with the protein surface, the system is typically discussed in terms of preferential hydration or preferential interactions. In the case of dry or almost dry solid-state formulations when water is almost fully removed by the drying procedure, the situation is different and can be discussed in terms of the two theories mentioned above. The drying of disaccharide–protein solution results in an amorphous phase with properties that are dependent on the properties of the individual components, i.e., disaccharides, proteins, and residual water and interactions between them.

One of the main characteristics of an amorphous or glassy state is the glass transition temperature (*T*_g_). It has been demonstrated that the disaccharide–protein mixtures obtained by using various drying methods exhibit a single glass transition.^1−4,7^ That implies the existence of a single amorphous phase, even though there is an example when a phase separation is thermodynamically favorable.^3^ The *T*_g_ of the protein–disaccharide mixture is higher than that of disaccharide and increases upon the addition of the protein within a certain concentration range. This has been interpreted in the literature as homogeneous mixing of glassy polymers where the resulting glass transition of the mixture can be described by using the Gordon–Taylor equation.^8^ Another possible explanation for the increase of the glass transition temperature could be the effect of confinement.^9^ The molecular weights and volumes of protein molecules are greater than those of disaccharides. At relatively high protein concentrations, the sugar domains can be visualized as confined between the protein molecules, which restricts the molecular motions.^1^

In addition to *T*_g_ measurements, spectroscopy studies of molecular interactions in disaccharide–protein solid mixtures have been reported.^2,3,7,10^ A major factor governing the molecular interactions in these systems is the formation of hydrogen bonds between protein and water, sugar and water, and protein and sugar. It has been shown that the stretching vibration of the asparagine side-chain stretching in vibrational spectra of proteins is sensitive to the presence of hydrogen bonds with sugars.^11^ The presence of hydrogen bonds between the sugar and the protein causes the frequency to shift to lower values with increasing sugar content.^7^ Also, the intensity of the α-helical band of lysozyme increases with increasing sucrose content,^7^ which suggests that the presence of sucrose changes the secondary structure. The location of residual moisture in freeze-dried protein–saccharide mixtures can be explored by infrared spectroscopy.^12^ At a low saccharide-to-protein ratio, water is located at the protein surface. At a high saccharide-to-protein ratio, high-frequency bands prevail, which indicates a higher number of sugar–water interactions than in the previous case. The size and shape of protein molecules in the solid state with and without excipients have been extensively studied by X-ray and neutron scattering techniques.^10,13−16^ In our previous work,^13^ we followed the structural changes in the lysozyme–water system using X-ray scattering experiments. We showed that the position of the protein–protein correlation peak, which appears between 2.09 and 2.61 nm^–1^, shifts to lower values upon hydration and interpreted this behavior as the swelling of the material. At water contents higher than 35 wt %, the form factor indicated an ellipsoidal shape of lysozyme molecules.^13^ Overall, lysozyme has a distorted structure in the dry state, which, upon swelling in water, transforms into the native ellipsoid shape.^13^

In this study, we have focused on the mixing behavior of binary and ternary systems: fully dehydrated mixtures of lysozyme–sucrose and lysozyme–sucrose–water formulations at low water contents. Lysozyme–sucrose freeze-dried formulations were chosen as a model system. Lysozyme is a stable protein with known size, shape, and hydration behavior, while sucrose is frequently used as a protein stabilizer in solid-state formulations. Using synchrotron small-angle X-ray scattering, differential scanning calorimetry (DSC), and gravimetric analysis (TGA), we have investigated the effect of temperature on the interactions of lysozyme in the amorphous state.

## Materials and Methods

### Materials

Lysozyme from chicken egg white (CAS number 12650-88-3, Lot SLBZ 2146, 96% purity) was purchased from Sigma-Aldrich. Lysozyme powder was purified by dialysis, as detailed below. Crystalline sucrose (CAS 57-50-1) > 99.5% purity was purchased from Sigma-Aldrich and used as received. Milli-Q purified water (ELGA, Purelab Flex) was used for all experiments.

### Methods

#### Dialysis

Lysozyme powder was dissolved in Milli-Q water to prepare a stock solution containing 4 wt % of the protein at 20 °C. After the complete dissolution of the powder, the stock solution was filtered through a 0.2 μm Acrodisc syringe filter to remove large-size aggregates before transferring to an Amicon ultracentrifugal filter tube (Merck) with a 3 kDa cutoff and a maximum initial sample volume of 15 mL. Water was exchanged several (approx. 8–10) times using centrifugation (Becker, 4000 g, 30 min). Lysozyme concentration after dialysis was measured at λ = 280 nm using a NanoDrop spectrophotometer ND1000 (NanoDrop technology). The solution was then adjusted to contain 2 wt % of the protein before lyophilization.

#### Freeze-drying

Lysozyme–sucrose formulations were prepared by lyophilization from aqueous solutions using a freeze dryer (Epsilon 2–6 LSCplus, Martin Christ GmbH, Germany) with a temperature-controlled shelf. The samples were prepared with lysozyme and sucrose at different ratios at a total content of 10 wt % of the solid material and were subsequently freeze-dried in 6 mL clear glass vials with a diameter of 22 mm (Schott, Germany) filled in with 2 mL of the solution. The vials were loaded at room temperature. During freezing, the temperature was lowered to −45 °C (0.2 °C/min) and held isothermally for 2 h. The samples were kept on the temperature-controlled shelf of the freeze dryer while the temperature was increased to 4 °C and the pressure lowered to 0.1 mbar. The primary drying was done in these conditions for 16 h. For the secondary drying, the temperature was increased to 20 °C in 1 h, while the chamber pressure was lowered to 0.01 mbar. After the ramp, the shelf was held isothermally for 3 h. At the end of the freeze-drying cycle, the chamber was filled with dry nitrogen, the vials were sealed and stored in a freezer at −20 °C until further analysis. No collapse was observed in the vials.

#### TGA

The residual water content was determined using thermal gravimetric analysis (Q500, TA Instruments). The samples with an amount of 5–10 mg of formulation were placed in open platinum pans, and these were, in turn, loaded into the sample compartment. TGA data were collected using a ramp of 10 °C/min between 25 and 200 °C. Each experiment was done 3 times, allowing for the calculation of the mean value and the standard deviation as presented in Table 1.

**Table 1:** Water Content, Water Activity, the Glass Transition Temperatures, and the Heat Capacity Step of Samples with Residual Moisture

Lys/suc	water content	*a*_w_ fast hydration	*a*_w_ slow hydration	*T*_g_, °C	Δ*C*_p_, J/gK	*T*_g_, °C	Δ*C*_p_, J/gK
wt %/wt %				(scan 1)	(scan 1)	(scan 2)	(scan 2)
0 100	1.2 ± 0.3	0.24	0.1^a^	43 ± 6	0.71 ± 0.06	Cryst	Cryst
10 90	1.4 ± 0.2			50 ± 4	0.65 ± 0.02	Cryst	Cryst
20 80	2.3 ± 0.1	0.27^b^		42 ± 1	0.63 ± 0.02	28 ± 1	0.38 ± 0.02
40 60	2.2 ± 0.3	0.29^b^		40 ± 2	0.47 ± 0.05	40 ± 1	0.49 ± 0.05
50 50	2.88 ± 0.03			31 ± 2	0.30 ± 0.03	32 ± 5	0.32 ± 0.02
100 0	4.3 ± 0.4	0.22^c^	0.1^c^				

a Calculated using data from ref (23).

b This work (sorption calorimetry).

c Calculated using data from ref (24).

#### DSC

DSC measurements were performed using DSC 1 (Mettler Toledo, Switzerland). Temperature calibration and heat flow calibration was done using indium. Glass transition temperature changes in heat capacities were determined using STARe Software following the ISO standard (ISO 11357-2:1999). An empty aluminum crucible was used as a reference.

The samples, as received after freeze-drying, were placed in 40 μm aluminum pans and hermetically sealed in a nitrogen atmosphere with a relative humidity of less than 5% and were subjected to program 1.

##### Program 1

Equilibration at 25 °C for 5 min, cooling 10 °C/min to −80 °C, isothermal for 5 min, heating 10 °C/min to 150 °C.

To obtain dry samples, the freeze-dried powders were additionally dried directly in a DSC pierced pan, as described below.

##### Program 2

The pans were pierced before the runs in this program. The runs of the program are as follows: equilibration at 25 °C for 5 min, heating 10 °C/min to 70 °C, isothermal for 20 min, cooling 10 °C/min to 0 °C, isothermal at 0 °C for 5 min, heating at 10 °C/min to 150 or 160 °C.

Experiments were performed 3 times for each concentration.

#### Sorption Calorimetry

Sorption calorimetric experiments were conducted at 25 °C in a 28 mm two-chamber sorption calorimetric cell inserted in a double-twin microcalorimeter. The samples under study were placed in the upper chamber, and pure water was injected into the lower chamber. The thermal energy released in the two chambers was monitored simultaneously. The water activity in the sorption experiments was calculated from the thermal power of vaporization of water in the lower chamber as described in ref (17). The partial molar enthalpy of the mixing of water was calculated according to ref (18).

#### SAXS

Small-angle X-ray scattering (SAXS) experiments were carried out at the CoSAXS station at the MAX IV synchrotron (Lund, Sweden).^19^ The samples were held in quartz capillaries (φ ∼ 1.5 mm) and hermetically sealed by super glue. The SAXS experiments were performed using an X-ray energy of 12.4 keV, corresponding to a wavelength of 0.1 nm. The two-dimensional SAXS images were recorded using the EIGER2 X 4M detector from Dectris (Baden-Daettwil, Switzerland), located at a distance of 2.31 m from the sample. The 2D SAXS patterns were corrected for transmission before orientally averaging to give intensity vs. *q*. The data were not rescaled to absolute intensities. A Linkam stage (HFSX 350, Linkam, UK) was used to control the heating/cooling procedure. The samples were heated from 25 to 145 °C, which is above the unfolding temperature of lysozyme at 137 °C as determined by DSC. The heating rate was set at 5 °C/min to obtain a data set with a temperature treatment comparable to that of the DSC measurements. The scattering curve of an empty capillary was recorded using the same setup. This scattering curve was subsequently subtracted from the scattering curves of the sample at different temperatures. The uncertainties of the scattering intensities of the resulting curves were obtained by error propagation.

## Results and Discussion

### Thermal Analysis

The residual water content and the thermal properties of the freeze-dried lysozyme–sucrose mixtures were studied by TGA and DSC.

### Samples with Residual Moisture

#### TGA Results

The water content of freeze-dried lysozyme samples is presented in Table 1. The residual moisture contents were different but, in all cases, less than 5 wt %, in agreement with previous studies of spray-dried and freeze-dried protein–disaccharides formulations.^20−22^ Pure freeze-dried sucrose had the lowest amount of water, and the addition of lysozyme increased the water content in the powders. Freeze-dried lysozyme without sucrose had the highest amount of residual water. All the samples in this study were freeze-dried in one batch, i.e., under the same drying conditions, which ensures the same water activity in the samples at the end of the drying process. Thus, the difference in water contents arises from the difference in water sorption at the same water activity.

The different residual moisture content in the freeze-dried samples can be explained by considering the water sorption isotherms for sucrose and lysozyme.^23,24^ The water activities corresponding to the residual moisture contents found in the samples are presented in Table 1. The water activity is about 0.1 for the corresponding water content for pure sucrose and pure lysozyme in slow hydration experiments and about 0.22–0.27 in fast hydration experiments in the sucrose–lysozyme mixtures and pure sucrose/lysozyme. This confirms that the residual water content is due to similar water activity.

The expected water activity at the end of the drying process might be calculated as a ratio of water vapor pressure (*P*_w_^0^) at the condenser temperature of −80 °C and at the shelf temperature of 20 °C at the secondary drying step. The predicted water activity of 2 × 10^–5^ is much smaller than the measured values (see Table 1). This discrepancy can be attributed to the water concentration gradients inside the samples or temperature gradients in the freeze-drying system (e.g., in the ice on the condenser).

#### DSC

**Figure 1:**
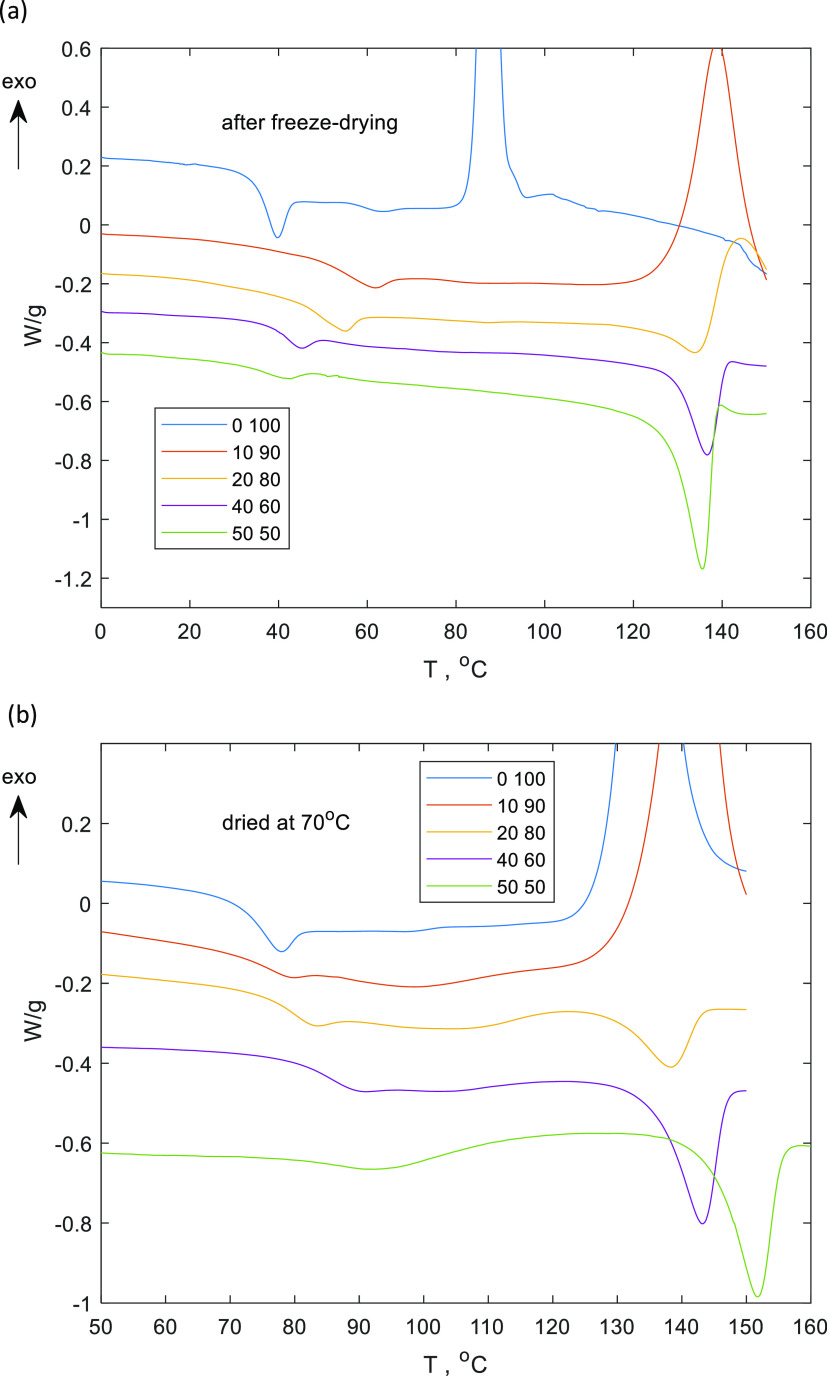
DSC heating scans (a) of the freeze-dried formulations with residual moisture (program 1) and the DSC heating scans (b) of freeze-dried formulations with drying at 70 °C (program 2). Different colors correspond with different lysozyme contents (the first number in the legend), e.g., 0 100 means 0 wt % lysozyme, 100 wt % sucrose.

Figure 1a shows typical DSC heating scans of lysozyme–sucrose samples at different protein–sugar ratios after freeze-drying that have been obtained using program 1 described above. Upon heating, freeze-dried sucrose undergoes glass transition, crystallization, and melting, where the melting is outside the temperature range covered in this study. The sample with 10 wt % of lysozyme and 90 wt % of sucrose behaves similarly to pure sucrose with respect to the glass transition step and the sucrose crystallization. However, the onset of crystallization shifts to higher values with increasing lysozyme concentration. The sample with a protein content of 20 wt % has an endodermic peak before the sucrose crystallization, which can be attributed to lysozyme denaturation. The samples with higher protein content also undergo glass transition and display a protein denaturation peak. The sucrose crystallization is not observed for samples with 40 and 50 wt % of lysozyme in the studied temperature range.

The glass transition temperature and the heat capacity step of lysozyme–sucrose formulations are given together with residual moisture data in Table 1. The glass transition of pure freeze-dried lysozyme is not visible in the DSC heating scans. It is well-known that for pure globular proteins (native or dried from the native state), the glass transition cannot be easily detected using calorimetry, while this is not the case for thermally denatured ones.^25^ The difficulties that arise in the detection of *T*_g_ of proteins can be explained in different ways. The protein glassy matrix has a broad distribution of relaxation times, which makes it impossible to detect the heat capacity step in DSC measurements, or the internal structure of the proteins hampers intermolecular dynamics needed for a glass transition.^25^

The studied mixtures have only one glass transition thermal event in the studied temperature range (25–150 °C), indicating only one glassy phase. All components in the system affect the *T*_g_ of the freeze-dried powder. Sucrose, as it has the highest weight fraction, is the main component of the glassy matrix. One can expect that the two other components present in the system influence the *T*_g_ of the matrix oppositely; water decreases,^26^ while lysozyme increases (see the next section of the Fully Dehydrated Lysozyme–Sucrose System). Since the water content increases with lysozyme content, the effect of lysozyme addition (which brings additional water to the system) on *T*_g_ is difficult to predict. The observed *T*_g_ of freeze-dried samples is the result of both effects (see Table 1).

From Table 1, it can be seen that the magnitude of the heat capacity step is correlated to the amount of sucrose present in the mixture. Lower sucrose content leads to lower values of the heat capacity step. We observe that the second scan of a sample containing 20 wt % of lysozyme shows a drop in both the glass transition temperature and the heat capacity step. This can be attributed to the partial crystallization of sucrose, which occurs during the first scan, and to the increase of the water content within the amorphous phase.

To analyze the mixing behavior in the lysozyme–sucrose binary system, the samples were additionally dried (the Program 2 section), and the results are presented in the next section.

### Fully Dehydrated Lysozyme–Sucrose System

We have investigated the thermal behavior of a lysozyme–sucrose mixture with minimal water content. The DSC pans were pierced before the experiments and dried at 70 °C, which is higher than the glass transition temperatures of the freeze-dried formulations (see Figure 1b and program 2).

The glass transition step, the sucrose crystallization, and the lysozyme denaturation thermal events were observed in the DSC curves of the dried samples (Figure 1b) as well as from the DSC curves of samples with residual moisture (Figure 1a). The sucrose crystallization peak is not visible in the case of 20% of lysozyme within the studied temperature range. In contrast, sucrose crystallizes when residual moisture is present (Figure 1a), i.e., the presence of water facilitates sucrose crystallization in the studied system. The DSC curves of the samples in the pierced pans (Figure 1b) exhibit a broad exothermic peak before the protein denaturation peak with a peak of about 120 °C. We attribute this broad event to the partial crystallization of sucrose.

**Figure 2:**
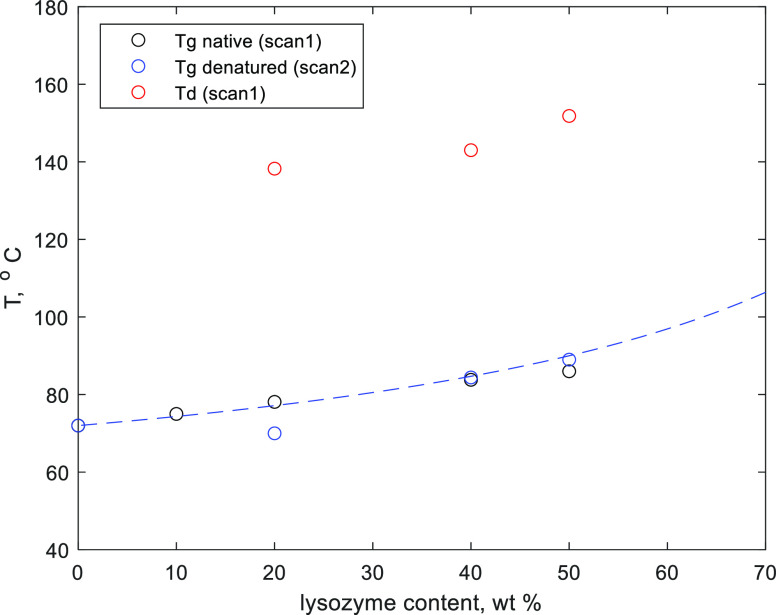
Glass transition temperature of the dry mixture as a function of the lysozyme content in the native and denatured states. The blue dashed line has been calculated according to eq 5 with *k* = 0.2, *and T*_d_ is the lysozyme denaturation temperature (see below).

The mixing behavior of the glassy multicomponent systems can be studied by DSC by analyzing the glass transition temperature and the heat capacity steps. The *T*_g_ values of the binary lysozyme–sucrose mixture increase upon increasing lysozyme concentration (Figures 1b and 2). As we have mentioned in the Introduction section, there are two possible explanations for this observation, the homogeneous mixing of two polymers^8^ or the effect of confinement on the glass transition.^9^ We will discuss both options below.

The obtained values of the *T*_g_ as a function of lysozyme content are presented in Figure 2 for native and denatured lysozyme–sucrose mixtures. Lysozyme is a high molecular weight compound; even though the glass transition temperature of native lysozyme is not observed in DSC, the value is expected to be substantially higher than for sucrose.

The composition dependence of *T*_g_ in binary systems is usually described by the Gordon–Taylor equation^8^
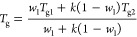
1where, in the case of the considered system, *T*_g1_ and *T*_g2_ would be the glass transition temperatures of pure sucrose and lysozyme, respectively, *w*_1_—the sucrose content, and *k*—the so-called Gordon–Taylor coefficient. However, the glass transition of native lysozyme is not present in the DSC scans obtained in this work. Nonetheless, eq 1 can be applied to the mixtures of denatured lysozyme and sucrose using the glass transition temperature of denatured lysozyme of 180 °C.^25^ The Gordon–Taylor equation is used here only for illustration of the trend at the low protein concertation range.

**Figure 3:**
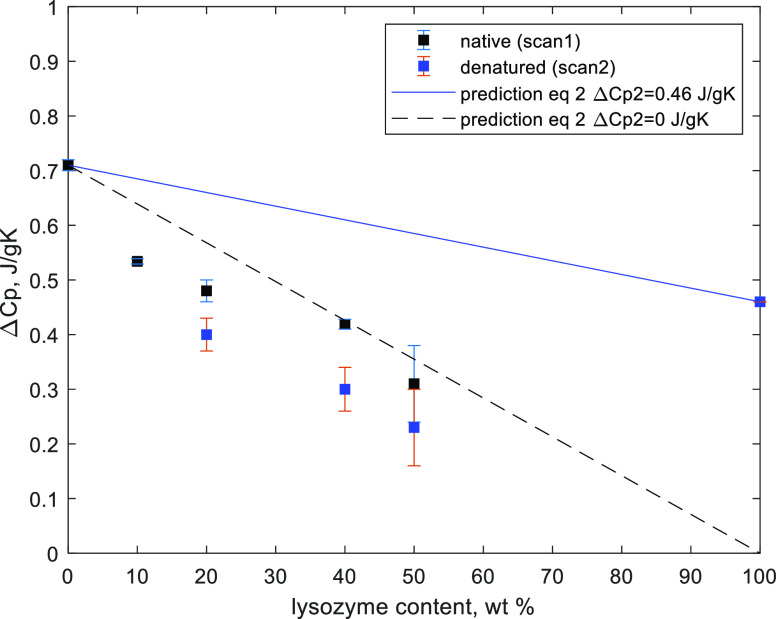
Heat capacity step in the fully dehydrated lysozyme–sucrose mixtures for native (scan 1) and denatured (scan 2) lysozyme. The predicted values are drawn as a blue solid (Δ*C*_p2_^0^ = 0.46 J/gK) and dashed black lines (Δ*C*_p2_^0^ = 0) (eq 2). Error bars represent the standard deviation of triplicate measurements.

The glass transition thermal event has another important characteristic—the heat capacity change between the glassy and liquid states. The heat capacity step of the lysozyme–sucrose mixture as a function of the lysozyme content is presented in Figure 3. The value of the heat capacity step decreases upon the increase of protein concentration. The heat capacity step (Δ*C*_p_) in a binary amorphous system, where both components take part in the glass transition, can be calculated as follows:
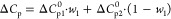
2where Δ*C*_p1_^0^ and Δ*C*_p2_^0^ are the heat capacity steps of pure sucrose (1) and lysozyme (2), respectively.

**Figure 4:**
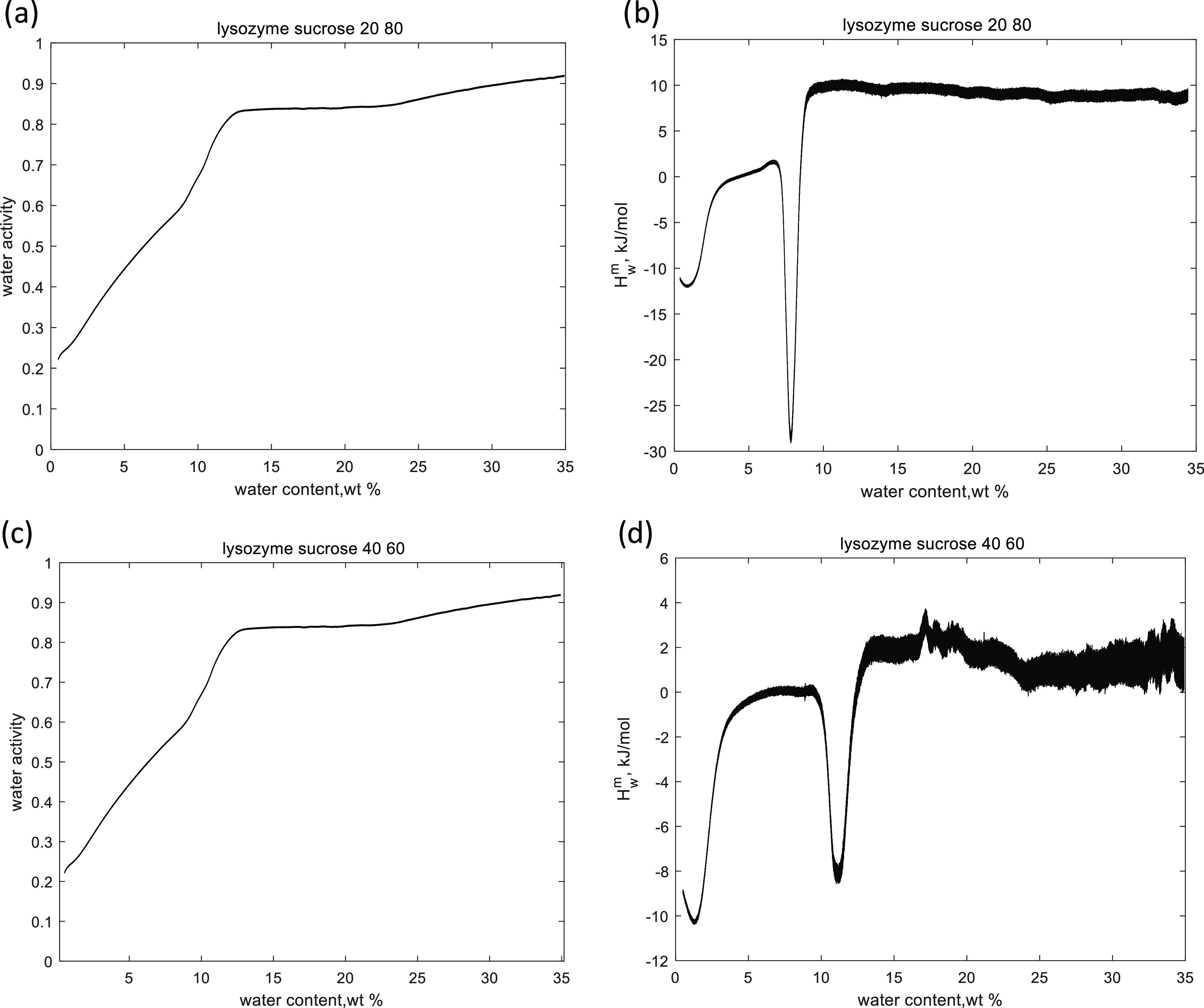
Sorption isotherms of lysozyme–sucrose mixtures with a lysozyme content of 20 wt % (a) and a lysozyme content of 40 wt % (c) and the partial molar enthalpies of mixing for lysozyme–sucrose formulations with a lysozyme content of 20 wt % (b) and a lysozyme content of 40 wt % (d).

The heat capacity step for glassy sucrose is 0.7 J/K/g.^23^ For denatured lysozyme, the heat capacity step is known to be 0.46 J/K/g.^25^ The experimental values in the binary system are lower than the values predicted from eq 2 (Figure 4). Moreover, they are somewhat lower even if Δ*C*_p2_^0^ is taken as zero (dashed line). Further decrease of the heat capacity change for scan 2 might be attributed to a partial sucrose crystallization. A mismatch of experimental data and eq 2 indicates that lysozyme does not undergo the glass transition with the sucrose glassy matrix. Thus, the increase of the glass transition temperature in the presence of lysozyme can be explained by the effect of confinement.^9^ The freeze-dried material has a composite-like structure; sucrose amorphous domains are trapped between lysozyme molecules that are in a “hard” rather than a flexible state at these conditions.

### Thermodynamic Parameters of Lysozyme Denaturation

The thermal denaturation of lysozyme is seen as an endothermic peak in the DSC scans. The denaturation temperature *T*_d_ is associated with the peak maximum, and integration of the area under the peak gives the calorimetric enthalpy (Δ*H*).

Figure 1 shows the denaturation peak of lysozyme at different sucrose concentrations both for samples with residual moisture as well as dried samples. The *T*_d_ in freeze-dried mixtures is higher than in lysozyme–water mixtures in the solid or liquid state.^16^ The calorimetric enthalpy (Table 2) has similar values to the enthalpy in aqueous solution.^16^ This confirms that in the solid sucrose matrix, lysozyme has a structure energetically similar to its native structure observed in an aqueous solution. One can see that the calorimetric enthalpy in the case of samples with residual moisture (Table 2) increases upon the increase of lysozyme content, while those samples also have a higher water content (c.f. Table 1). Therefore, water evaporation is one of the possible reasons for the enthalpy increase.

**Table 2:** Thermodynamic Parameters Denaturation Temperature *T*_d_ and Calorimetric Enthalpy Δ*H* for the Denaturation of Lysozyme at Different Concentrations^a^

	residual moisture, sealed pan		dried, pierced pan	
lysozyme content, wt %	*T*_d_ (°C)	Δ*H* (kJ/mol), mol of lysozyme	*T*_d_ (°C)	Δ*H* (kJ/mol), mol of lysozyme
20	134.1 ± 0.4	470 ± 30	138.2 ± 0.1	500 ± 10
40	137 ± 1	670 ± 50	143.0 ± 0.1	522 ± 5
50	135 ± 1	770 ± 40	151.8 ± 0.1	459 ± 8

a Each value is the average of 3 measurements and the standard deviation of triplicate measurements is shown.

The denaturation temperature of lysozyme does not show a clear dependence on protein content for samples with residual moisture (see Table 2). In contrast, in the case of fully dehydrated samples, the denaturation temperature shifts to higher values with increasing lysozyme content (see Table 2 and Figure 2). To understand that, we consider a mechanism of protein denaturation.

The DSC peak of lysozyme denaturation has a slightly asymmetric shape (c.f. Figure 1), which indicates an irreversible process, and the kinetic approach should be used to analyze experimental results.^27^ It was shown in our previous study^16^ that water activity (*a*_w_) has a big impact on lysozyme denaturation in the solid state. While solvation can stabilize the native state of protein molecules, it can also stabilize the unfolded molecules. Moreover, since in the unfolded state, one can expect more protein–solvent contacts due to a less compact structure, more solvent is needed for the stabilization of the unfolded structure. Hence, irreversible lysozyme denaturation processes facilitated by the presence of water or sucrose can be written as follows:
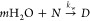
3
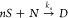
4where *S* is sucrose, *m* and *n* are the number of water or sucrose molecules involved in the reaction, respectively, *N* and *D* are the native and denatured protein, respectively, *k*_w_ is the reaction constant in the case of water, and *k*_s_ is the reaction constant in the case of sucrose.

The process of lysozyme denaturation in water or sucrose can be formally described by similar equations
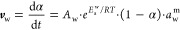
5
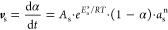
6where *v*_w_ and *v*_s_ are the conversion rates, respectively, α is the degree of conversion from the native to the denatured state, *E*_a_^w^ and *E*_a_^s^ are the activation energies of the reaction, respectively, *A*_w_ and *A*_s_ are pre-exponential factors, respectively, and *a*_s_ is the thermodynamic activity of sucrose.

In the case of lysozyme–sucrose samples with residual moisture, both reactions according to eqs 3 and 4 occur. The reaction rate with water (*v*_w_, eq 5) depends on water activity, which is shown in Table 1. As the water activity remains constant at different lysozyme contents in the mixtures, one can expect similar reaction rates for samples with residual moisture. The maximum reaction rate corresponds with *T*_d_ at the DSC denaturation peak. The denaturation temperature *T*_d_ does not show any dependence on the lysozyme content (c.f. Table 2) since the samples exhibit constant water activity. The reaction, according to eq 5 accounts, for the main contribution to the denaturation mechanism in samples with residual moisture.

In dry samples, the water activity is close to 0 and only the reaction according to eq 4 occurs. Although the sucrose activities in the mixtures with lysozyme are not known quantitatively, they correlate with the sucrose concentration. Therefore, higher sucrose concentrations require lower temperatures for the same reaction rate to occur.

### Water Sorption Behavior

The water sorption isotherms and corresponding hydration enthalpies for two freeze-dried formulations are presented in Figure 4. The initial hydration enthalpy of the samples is exothermic, which was also observed for pure sucrose and pure lysozyme.^23,24^ A step in both enthalpy curves around a water content of 2 wt % (*a*_w_ = 0.2–0.3) is the water-induced isothermal glass transition. The next region is the mixing of the metastable liquid with water (*H*_w_^m^ ≈ 0), followed by an exothermic peak of hydration-induced sucrose crystallization. The sample with 20% of lysozyme crystallizes at a water content of 8 wt % (*a*_w_ = 0.45–0.5), and the sample with lysozyme of 40 wt % crystallizes at a higher water concentration of 11 wt %. After the sucrose crystallization event, there is the dissolution of the sucrose crystal region in both cases. The lysozyme (40 wt %)–sucrose sample also shows a dilution region that starts around 23 wt % of water (as seen from the rising water activity level and low hydration enthalpy values).

The hydration behavior of the studied formulations is similar to freeze-dried sucrose.^23^ In other words, the isothermal phase transitions in the lysozyme–sucrose mixtures, namely, the glass transition and the sucrose crystallization, match the transitions occurring in sucrose upon the increase of water content, while pure lysozyme shows different responses to hydration.^24^ We conclude that the properties of sucrose define the hydration properties of the lysozyme–sucrose mixtures presented in this study.

### SAXS Results

The structure of the lysozyme–sucrose freeze-dried samples with residual moisture content at 25 °C and upon heating to 145 °C was studied by means of SAXS experiments. The scattering data cover a *q*-range of 0.1–10 nm^–1^.

### Overview of the Scattering Pattern

**Figure 5:**
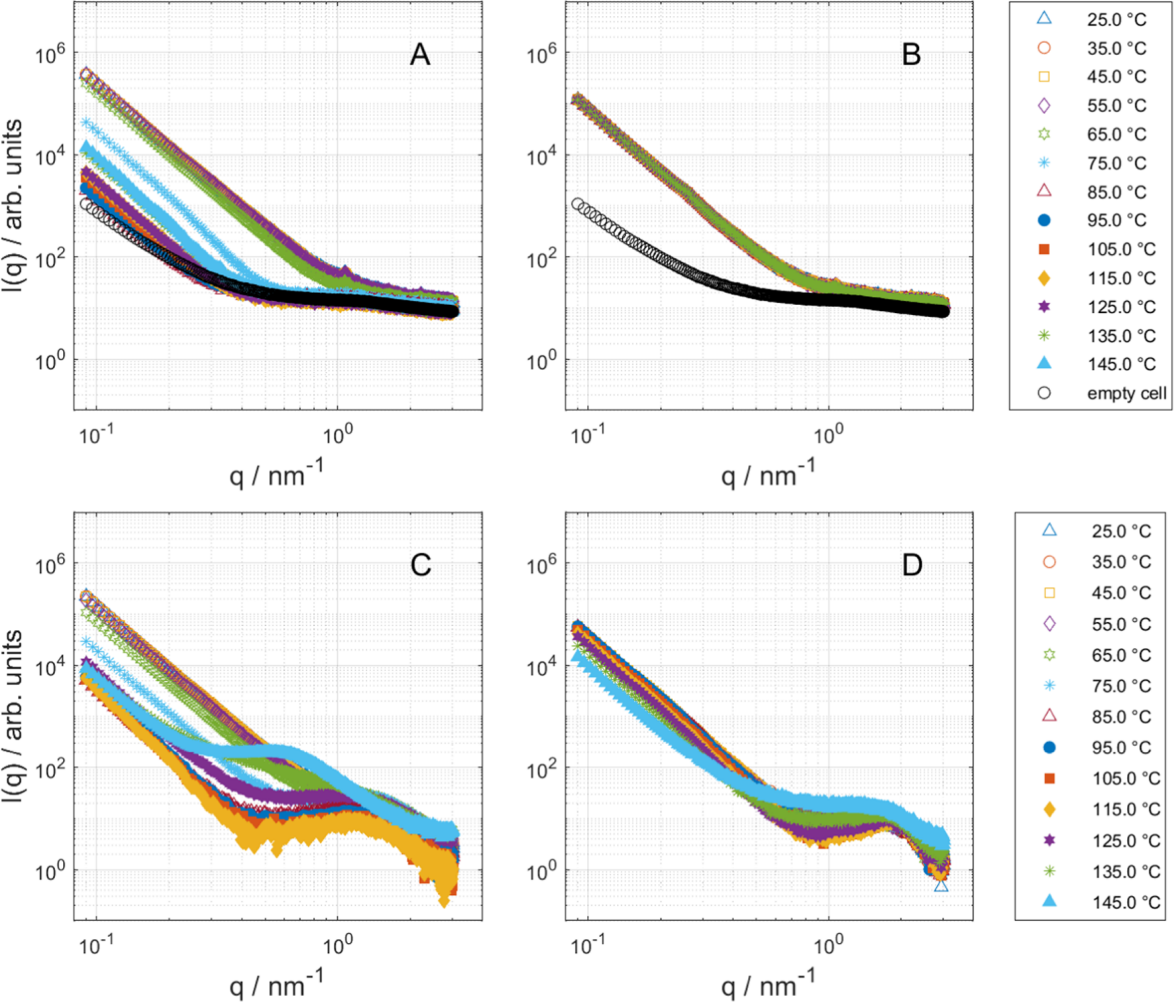
Scattering curves recorded between 25 and 145 °C. (A) Pure sucrose. (B) Pure lysozyme. (C) Lysozyme–sucrose with 20.0 wt % lysozyme. (D) Lysozyme–sucrose with 40.0 wt % lysozyme. In plots (A) and (B), the background was not subtracted and the scattering curve of the empty capillary is shown for comparison, while plots (C) and (D) show the scattering curves of the mixtures after the subtraction of the empty cell.

An overview of the scattering patterns of lysozyme, lysozyme–sucrose mixtures, and sucrose is shown in Figure 5. Since the samples are amorphous powders, the scattering curves exhibit a power law dependence at *q* < 1 nm^–1^ with a slope of around −4 (Figure 5). A slope of −4, in this case, results from the scattering at the solid–air interface of the powder. The intensity of scattering at low *q* is strongly temperature-dependent for all samples except pure lysozyme. Several correlation peaks are observed within the studied *q*-range. The correlation peak at *q* = 2.5 nm^–1^ in pure lysozyme (Figure 5 B) corresponds to the protein–protein distance of 2.5 nm, which also suggests that the average shape of lysozyme molecules is substantially distorted compared to the native conformation.^13^ The SAXS patterns of lysozyme mixtures with sucrose are different and display a broad peak, previously reported in the literature.^15^ We hypothesize that in these mixtures, lysozyme molecules retain shapes close to those observed in aqueous solutions since sucrose molecules can fill the space between protein molecules and also provide hydrogen bonds for the protein surface groups. To further investigate this hypothesis, we performed modeling of the SAXS data assuming ellipsoidal shapes of lysozyme molecules, which is close to the shape observed in liquid water.^13^

### Modeling of SAXS Data

**Figure 6:**
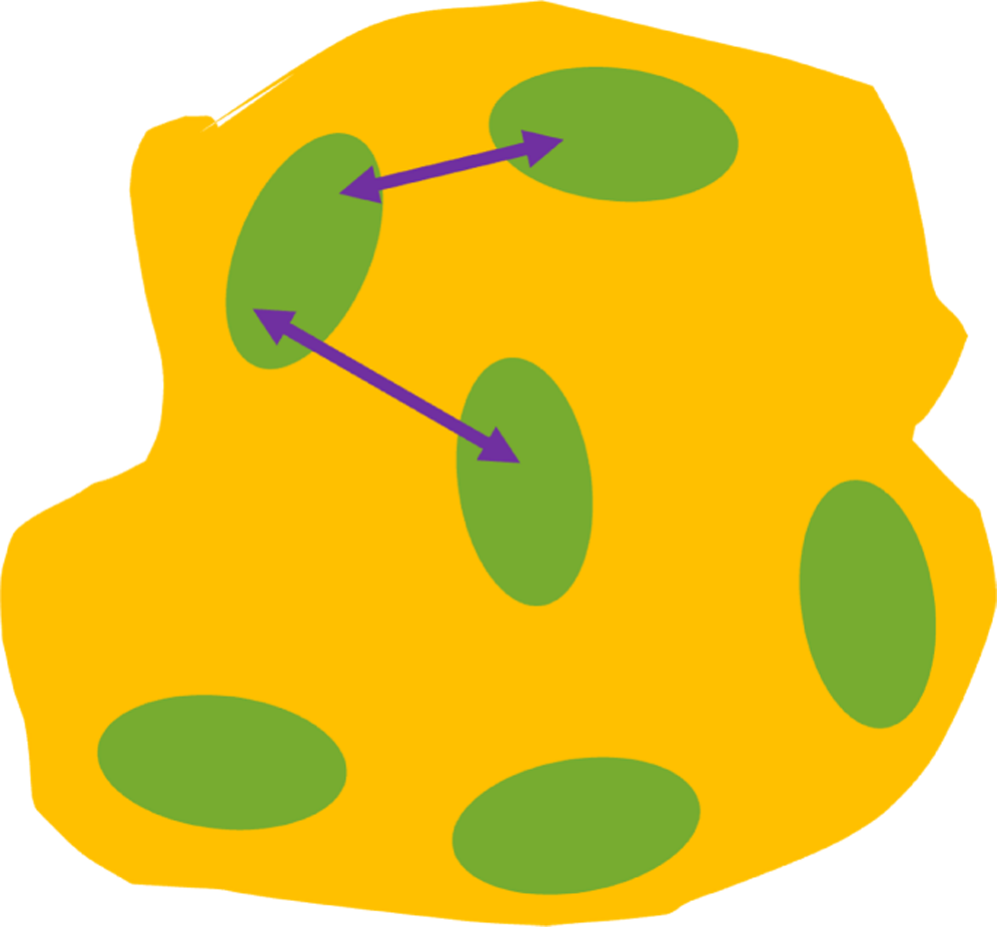
Lysozyme (green ellipsoids) is embedded in a sucrose grain (yellow). The arrows indicate the interaction between the ellipsoids.

Figure 6 shows an illustration of the model that is used to describe the scattering curves. The protein molecules are modeled with the form factor *P*(*q*)^28,29^ of ellipsoidal particles with constant equatorial radii (*r*_*x*_ = *r*_*y*_) and polar radius *r*_*z*_. These ellipsoids are embedded in a sucrose matrix. The interactions between the ellipsoids are taken into account with the structure factor *S*(*q*)^30^ for monodisperse, hard spheres, with the volume fraction η and effective radius *r*_eff_. In order to account for the nonspherical shape of lysozyme, the so-called decoupling approximation is applied.^28,29^

The sucrose matrix is represented as large grains without assuming any specific shape. These grains cause the scattering intensity to decay according to a power law, and at low *q*-values, this decay will be the dominating contribution to the curve. The form factor and the interactions of lysozyme will become the dominating contribution to the scattering curves at higher *q*-values. A constant background is applied to the model. Thus, the model is as follows:
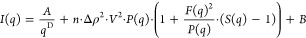
7

The first term on the right-hand side in eq 7 represents the power law. The power law amplitude *A* is proportional to the specific surface of the grainy sucrose matrix, and the power law exponent *D* is a measure of the surface roughness. The second term of eq 7 represents the shape of lysozyme and the interactions between them. From left to right, the variables are the number density *n*, the contrast Δρ, the volume *V* of lysozyme, the orientationally averaged scattering intensity *P*(*q*), and the orientationally averaged scattering amplitude *F*(*q*) of the ellipsoids. The interactions between different lysozyme molecules are accounted for by the structure factor *S*(*q*). *B* is the constant background.

The contrast Δρ and the number density of lysozyme in sucrose could be estimated; however, one of these parameters should be unconstrained during fitting and, with the scale A for the first term in eq 7, account for the fact that the scattering intensities are not rescaled to absolute intensities. The lysozyme number density in the lysozyme–sucrose mixtures is calculated using densities of ρ(lysozyme) = 1.4 g/cm^3^^13^ and ρ(sucrose) = 1.5 g/cm^3^^31^ and the molar mass of *M*(lysozyme) = 14.3 kDa [2].^29^ The number density of lysozyme can be estimated to be *n* = 1.26 × 10^19^ cm^–3^ and *n* = 2.51 × 10^19^ cm^–3^ for the mixtures containing 20.0 and 40.0 wt % of lysozyme, respectively.

The volume *V* of the ellipsoids reads as
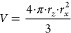
8The orientationally averaged scattering amplitude *F*(*q*) is given by the following equation
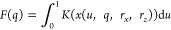
9
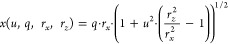
10
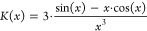
11The form factor *P*(*q*) is calculated as follows:
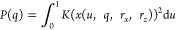
12

Integration of eqs 9 and 12 is shown in the SI.

Finally, the equation to calculate the hard-sphere structure factor reads as
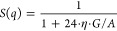
13where
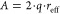
14

The parameter *G* depends on the volume fractions of ellipsoids η. Its definition and more detailed information on its calculation are presented in the SI in eqs E2–E10.

With eq 7, the data fitting includes eight unconstrained parameters: the power law amplitude *A*, the power law exponent *D*, the equatorial radius *r*_*x*_ and polar radii of the ellipsoid *radius r*_*z*_, the contrast Δρ, the volume fraction of the ellipsoids η and their effective radius *r*_eff_, and a constant background *B*. The calculated fitting parameters for all concentrations and temperatures are summarized in Tables S1 and S2 in the SI (an example of the temperature dependence of these parameters is also shown in Figure 8). Examples of SAXS curves fitting for three different temperatures and two concentrations are presented in Figure S1. These examples demonstrate that the model provides a reasonable fit to the experimental data; moreover, the obtained parameters such as ellipsoid radii and volume fractions are physically meaningful. For example, the ellipsoid radii at temperatures below denaturation are close to the ellipsoid parameters obtained in the liquid.^16^ This suggests that the protein molecules in the solid state in the presence of sucrose retain shapes close to their native shape (observed in aqueous solutions). A detailed discussion of the obtained model parameters is presented in the following sections. Moreover, based on the temperature dependencies of the obtained fitting parameters, we will discuss the two main processes that occur upon heating the protein samples: the glass transition and unfolding.

### Effect of the Glass Transition

Sucrose heating experiments display a strong reduction of the scattering intensity above 55 °C (Figure 5a). This corresponds to a decrease in the surface area of the freeze-dried particles and correlates with the glass transition temperature in DSC scans (see Table 1). In contrast, the SAXS patterns of freeze-dried lysozyme do not significantly change within the temperature range that we have investigated (c.f. Figure 5b). As mentioned before, dry lysozyme does not undergo a glass transition in the studied temperature range.

**Figure 7:**
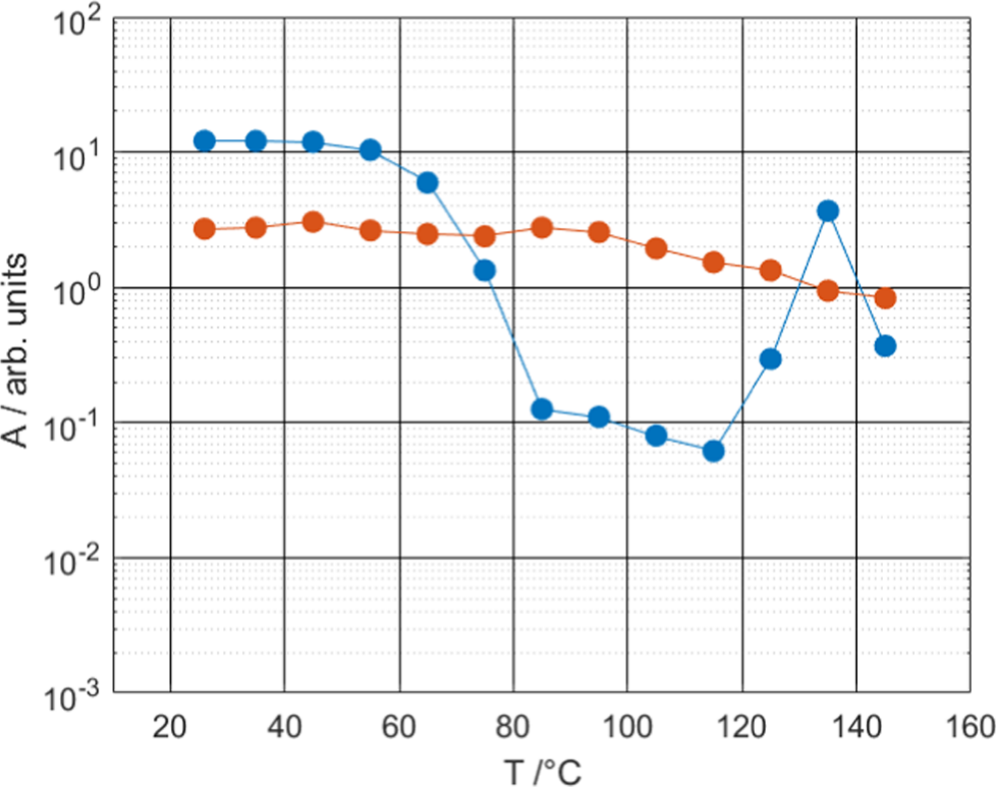
Power law amplitude as a function of temperature for the lysozyme–sucrose mixture with 20.0 wt % lysozyme (blue) and 40.0 wt % lysozyme (red).

The capillaries for SAXS measurements were hermetically sealed before the experiments, so we assume that there is no water loss during experiments. The lysozyme–sucrose mixtures display a decrease in scattering intensity upon heating, which is convenient to characterize by the power law amplitude *A*; see Figures 5c,d and 7. A remarkable difference between the two samples is that the power law amplitude of the sample with 20 wt % lysozyme undergoes an oscillation that covers two orders of magnitude, while the amplitude of the higher protein content sample decreases only by a factor of 3. Because a change in the power law amplitude is a measure of the surface area, we can infer that there is a change in the surface area of the sample with the lower lysozyme content at around 75 °C, a temperature close to the glass transition temperature. Since the power law exponent is around 4.0 (or even higher), the transition should be considered in terms of surface area rather than roughness. The increase of power law amplitude above 115 °C can be coupled to the onset of sucrose crystallization above this temperature.

In the case of the 40 wt % sample, a much lower decrease of the power law amplitude *A* is observed. This is probably related to the fact that lysozyme molecules do not undergo a glass transition together with sucrose, as observed in the thermal analysis (see the Thermal Analysis section above). The sucrose may be thought of as providing a scaffold that mechanically stabilizes the lysozyme protein.

Interestingly, in the majority of the considered cases, the power law exponent *D* is slightly higher than 4.0 (Tables S1 and S2), and this result seems to be robust because the calculated error is typically much lower than the deviation from 4.0. This observation might sound surprising because, in terms of surface fractals, the maximum exponent value can be 4.0, which corresponds to a smooth 2D surface, while lower values correspond to rough surfaces. A possible explanation for this experimental result can be found in the idea that so-called “fuzzy boundaries,” where the density does not change abruptly at the interface but rather decays continuously, can result in power law exponents greater than 4.0.^32^ This continuous change of density could be caused by preferential adsorption/accumulation of lysozyme molecules at the particle′s surfaces. Since lysozyme and sucrose have different densities, the accumulation of lysozyme at the interface eliminates the stepwise change of density required for obtaining the exponent of 4.0 for smooth surfaces. That explanation is in line with previous analysis^33^ of frozen lysozyme solutions. It was shown that the interface is enriched by lysozyme.

### Size of Lysozyme Molecules and Their Unfolding in Sucrose

The model for interpreting the SAXS data relies on the size characteristics of lysozyme molecules when calculating both the form and structure factors. The form factor uses the equatorial radius *r*_*x*_ and the polar radius *r*_*z*_ of the ellipsoid, which for further comparison, can be combined to define the equivalent radius *r*_eqv_
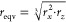
15

The structure factor ignores any anisotropy in the shape of the interacting particles and instead gives an effective radius *r*_eff_, which can be further interpreted in terms of effective volume *V*_eff_
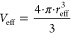
16

**Figure 8:**
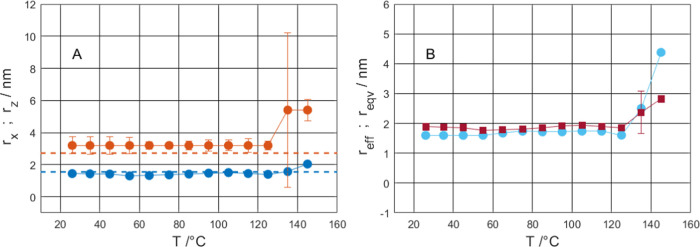
Change of radii with increasing temperature for the sample with 20.0 wt % lysozyme with *r*_*x*_ (blue solid circle) and *r*_*z*_ (red solid circle) (A) and *r*_eff_ (sky-blue solid circle) and *r*_eqv_ (brownish-red solid square) (B). The dashed lines indicate the dimensions of the ellipsoid from solution scattering.^13^

The temperature-dependent change of the equatorial radius *r*_*x*_ and the polar radius *r*_*z*_ of the ellipsoid, as well as the change in the volume fraction η and effective radius r_eff_, are plotted in Figures 8, S2, and S3 (in SI).

The equatorial radius *r*_*x*_ and the polar radius *r*_*z*_ of lysozyme in the sucrose matrix are in good agreement with the size and shape of lysozyme in solution^13^ up to 125 °C (Figure 8A).

The fact that lysozyme has similar structural features in a liquid aqueous solution and in a solid glassy sucrose matrix indicates that in the absence of water, amorphous sucrose provides an environment needed for maintaining the native structure. When neither water nor sucrose is present in the system, lysozyme molecules lose their native structure due to the necessity to continuously feel the space.^13,34^ Moreover, due to higher viscosity and higher *T*_g_ of sucrose, it is able to stabilize protein molecules up to higher temperatures compared to water.

At higher temperatures, the polar radius dramatically increases, which correlates well with the denaturation temperature observed in DSC (134–137 °C). Although the large error bars at high temperatures (together with the unexpected decrease of volume fraction η) suggest that the presented model is not optimal for denatured lysozyme, the volume expansion of lysozyme is in line with a typical idea about structural changes expected upon unfolding. In particular, the protein structure becomes less compact, increases in size, and opens up more contact with the solvent/matrix. This also implies that the solvent (sucrose in this case), to a certain degree, penetrates into the protein structure. This can be seen as an analogy with protein unfolding in an aqueous environment, where the lysozyme molecule increases in size and captures water inside.^13^

The effective radius *r*_eff_ obtained from the structure factor is in good agreement with the equivalent radius from the form factor (see Figure 8b) and shows the same trend with temperature, which further supports the applicability of the model and interpretations presented above. The protein volume fraction η obtained from the structure factor (Tables S1 and S2) is somewhat higher than the value expected from the density arguments for the native protein. This can be explained by the idea that the distribution of protein in the sucrose matrix can be nonuniform, and the regions with higher concentrations of protein can contribute more strongly to the scattering intensity. This observation is in agreement with power law exponent values higher than 4.0, as discussed in the previous section.

According to the SAXS findings presented here, lysozyme molecule size and shape in the glassy sucrose matrix correspond to those in aqueous solutions. This agrees with our DSC results, showing that the calorimetric enthalpy of lysozyme denaturation in a glassy sucrose matrix is comparable with unfolding enthalpy in an aqueous solution (even though the denaturation occurs at different temperatures). These observations support the idea that in the solid state, sucrose preserves the native structure of lysozyme, which is otherwise altered.^13^ In this regard, one can recall two theories of protein stabilization by sugars in the solid state: water replacement and vitrification. The similarities in the shape, size, and energy of denaturation in glassy sucrose and aqueous liquid strengthen the *w*ater replacement theory. Besides, the effect of water activity/water content on the denaturation temperature in glassy sucrose supports the vitrification theory. We suggest that these theories do not exclude each other and can be considered together.

## Conclusions

Here, we present a study on the interactions between sucrose, lysozyme, and water by DSC, sorption calorimetry, and small-angle X-ray scattering. We conclude that solid amorphous freeze-dried lysozyme, sucrose, and lysozyme–sucrose formulations have different residual moisture contents when prepared using the same procedure. The addition of sucrose lowers the residual moisture content of the lysozyme formulation because sucrose has lower equilibrium water content at the same water activity. Sorption calorimetry experiments show that the hydration behavior of sucrose rather than lysozyme dominates the hydration profile of lysozyme–sucrose formulations. Isothermal water-induced glass transition and sucrose crystallization are observed in lysozyme–sucrose mixtures.

In temperature-resolved SAXS and DSC experiments, glass transition and thermal denaturation of lysozyme were investigated. The addition of lysozyme increases the *T*_g_ of the lysozyme–sucrose system due to the confinement of sucrose between lysozyme molecules that do not undergo the glass transition with the sucrose matrix. The enthalpy of lysozyme denaturation in solid mixtures with sucrose has similar values in comparison with the aqueous solutions of this protein. This is an indirect confirmation of the fact that in the sucrose glassy matrix, the protein has a native structure. In line with the DSC results, the modeling of SAXS data shows that in the glassy sucrose matrix below denaturation temperatures, lysozyme molecules keep the same structure as in an aqueous solution. This is in stark contrast to the case of pure lysozyme without excipients, where it exhibits a different structure. Unlike the reversible thermal unfolding of lysozyme in aqueous solutions, its denaturation in sucrose is irreversible and occurs at much higher temperatures due to the slow kinetics. The residual moisture in freeze-dried formulations decreases the denaturation temperature of lysozyme.

## Supporting Information Available

Additional details of the SAXS model and SAXS data fitting results: equations S1–S10, figures S1–S3, and tables S1 and S2 (PDF)
