# Supplementary material for: Lysozyme–Sucrose Interactions in the Solid State: Glass Transition, Denaturation, and the Effect of Residual Water
Source: Mol Pharm. 2023 Aug 9;20(9):4664–75. doi: 10.1021/acs.molpharmaceut.3c00403 (PMC10481396; doi:10.1021/acs.molpharmaceut.3c00403)
Supplement: Supplementary file 1 — mp3c00403_si_001.pdf [file mp3c00403_si_001.pdf]

Lysozyme-sucrose interactions in the solid-state: the glass transition, denaturation and the effect of residual water

Supporting information

Ekaterina Bogdanova[1,2], Sebastian Lages[1,2,4], Tuan Phan-Xuan[1,2,4,**], Md. Arif Kamal[1,2,5], Ann Terry[4], Anna Millqvist Fureby[3] and Vitaly Kocherbitov[1,2,*]

[1] Biomedical Science, Malmö University, Malmö, SE-20506, Sweden;

[2] Biofilms research center for Biointerfaces, Malmö, SE-20506, Sweden;

[3] RISE Research Institutes of Sweden, Stockholm, SE-114 86, Sweden;

[4] MAX IV Laboratory, Lund University, Lund, SE-22484, Sweden;

[5]Division of Physical Chemistry, Lund University, Box 124, Lund, SE-221 00, Sweden

**Current address: Diabetes API Manufacturing Development, Novo Nordisk A/S, DK-4400, Kalundborg, Denmark

*- Corresponding author. E-mail: vitaly.kocherbitov@mau.se

The integrals in eq. 9 and eq. 12 are evaluated numerically. In literature, the so-called beta-correction β*(q)* is also frequently found:

$$\beta(q) = \frac{F(q)^2}{P(q)} = \frac{F(q)\cdot F(q)}{P(q)} = \frac{\langle F(q)\rangle\cdot\langle F(q)\rangle}{\langle F^2(q)\rangle} = \frac{\left(\int_0^1 K(x(u,q,r_x,r_z))du\right)\cdot\left(\int_0^1 K(x(u,q,r_x,r_z))du\right)}{\int_0^1 K(x(u,q,r_x,r_z))^2 du} =$$

$$\frac{\left(\int_0^1 K(x(u,q,r_x,r_z))du\right)^2}{\int_0^1 K(x(u,q,r_x,r_z))^2 du} = \frac{\langle F(q)\rangle^2}{\langle F^2(q)\rangle} \qquad \text{(S1)}$$

The hard-sphere structure factor ($S(q)$) has value G that was calculated as follows

$$G = G_1 + G_2 + G_3 \qquad \text{(S2)}$$

$$G_1 = \alpha\cdot\frac{a - A\cdot c}{A^2} \qquad \text{(S3)}$$

$$G_2 = \beta\cdot\frac{2\cdot A\cdot a + (2 - A^2)\cdot c - 2}{A^3} \qquad \text{(S4)}$$

$$G_3 = \gamma\cdot\frac{4\cdot[(3A^2 - 6)\cdot c + (A^3 - 6A)\cdot a + 6] - A^4\cdot c}{A^5} \qquad \text{(S5)}$$

$$\alpha = \frac{(1 + 2\cdot\eta)^2}{(1 - \eta)^4}$$

(S6)

$$\beta = -6\cdot\eta\frac{(1 + \eta/2)^2}{(1 - \eta)^4} \qquad \text{(S7)}$$

$$\gamma = \frac{\eta\cdot\alpha}{2} \qquad \text{(S8)}$$

$$a = sin(A) \qquad \text{(S9)}$$

$$c = cos(A) \qquad \text{(S10)}$$

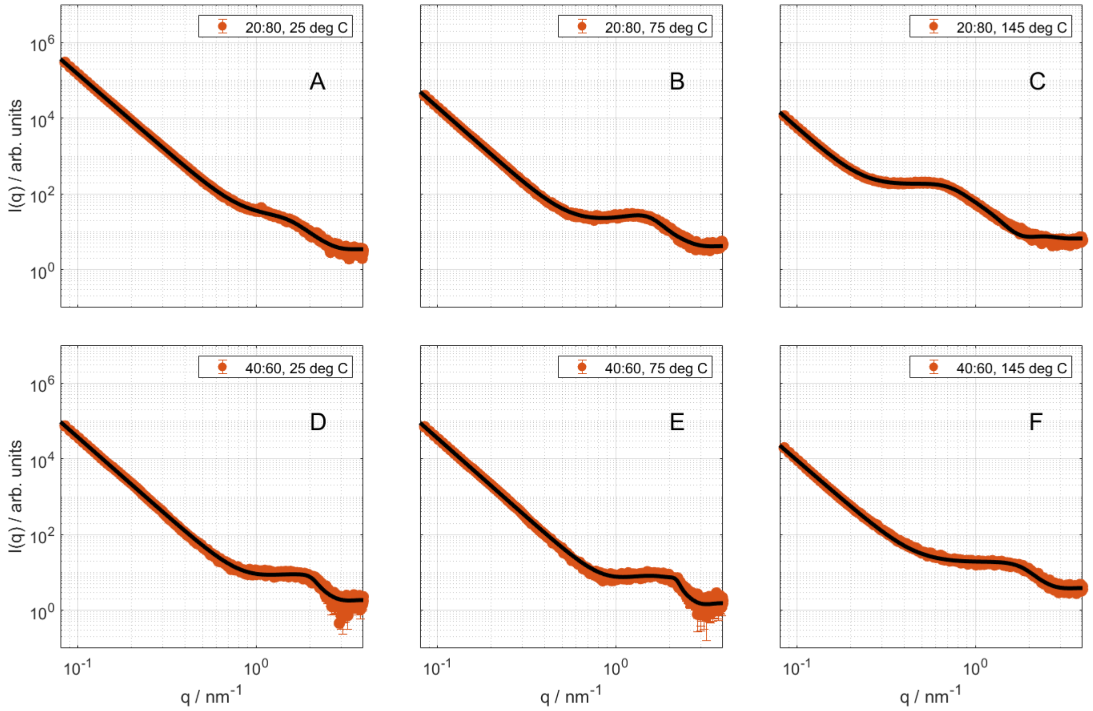

Figure S1. Examples of SAXS data fitting. Red is data, black- resulted fitting curve.

Table S1: Modelling results for the scattering curves of the mixtures with 20.0 wt-% lysozyme. The temperature range is 25 °C (1st row) to 145 °C (last row), in steps of 10 °C. The confidence intervals of *±1σ* are listed as well. If the confidence interval < 0.005, a value of 0.00 is listed. The power law amplitude *A*, the contrast *Δρ* and the constant background *B* are given in arbitrary units because the experimental scattering intensities could not be obtained on absolute scale. The power law exponent *D* and the volume fraction *η* are dimensionless quantities.

***T*°C**	***A***	***D* / []**	***Δρ***	***r_x* / nm**	***r_z* / nm**	***r_{eff}* / nm**	***η* / []**	***B***
**25**	12.09 ± 0.05	4.08 ± 0.00	3.4 ± 0.2	1.45 ± 0.04	3.2 ± 0.5	1.60 ± 0.04	0.22 ± 0.01	3.6 ± 0.1
**35**	12.07 ± 0.06	4.08 ± 0.00	3.4 ± 0.2	1.43 ± 0.04	3.2 ± 0.5	1.60 ± 0.04	0.22 ± 0.01	3.5 ± 0.1
**45**	11.89 ± 0.05	4.08 ± 0.00	3.4 ± 0.2	1.42 ± 0.04	3.2 ± 0.6	1.60 ± 0.04	0.22 ± 0.01	3.6 ± 0.1
**55**	10.38 ± 0.04	4.07 ± 0.00	3.6 ± 0.2	1.31 ± 0.05	3.2 ± 0.5	1.60 ± 0.03	0.22 ± 0.01	3.1 ± 0.2
**65**	5.96 ± 0.03	4.07 ± 0.00	4.1 ± 0.1	1.33 ± 0.03	3.2 ± 0.2	1.68 ± 0.02	0.25 ± 0.01	3.8 ± 0.1
**75**	1.34 ± 0.02	4.17 ± 0.00	4.04 ± 0.08	1.37 ± 0.02	3.2 ± 0.2	1.74 ± 0.02	0.28 ± 0.01	3.9 ± 0.1
**85**	0.13 ± 0.01	4.42 ± 0.02	2.87 ± 0.09	1.42 ± 0.03	3.2 ± 0.3	1.72 ± 0.02	0.27 ± 0.01	2.24 ± 0.09

**95**	0.11 ± 0.00	4.54 ± 0.02	2.39 ± 0.09	1.47 ± 0.04	3.2 ± 0.3	1.72 ± 0.03	0.27 ± 0.01	1.62 ± 0.08
**105**	0.08 ± 0.00	4.63 ± 0.01	2.12 ± 0.08	1.51 ± 0.04	3.2 ± 0.3	1.74 ± 0.03	0.28 ± 0.01	1.24 ± 0.07
**115**	0.06 ± 0.00	4.76 ± 0.02	2.0 ± 0.1	1.45 ± 0.05	3.2 ± 0.4	1.74 ± 0.04	0.28 ± 0.02	0.94 ± 0.08
**125**	0.30 ± 0.01	4.40 ± 0.01	3.8 ± 0.1	1.40 ± 0.02	3.2 ± 0.3	1.61 ± 0.02	0.22 ± 0.01	4.0 ± 0.1
**135**	3.7 ± 0.4	3.12 ± 0.03	2.2 ± 0.1	1.57 ± 0.05	5.4 ± 0.2	2.50 ± 0.07	0.15 ± 0.04	5.5 ± 0.3
**145**	0.37 ± 0.04	4.18 ± 0.02	1.9 ± 0.1	2.04 ± 0.01	5.4 ± 0.7	4.38 ± 0.06	0.15 ± 0.01	5.8 ± 0.1

Table S2: Modelling results for the scattering curves of the mixtures with 40.0 wt-% lysozyme. The temperature range is 25 °C (1st row) to 145 °C (last row), in steps of 10 °C. The confidence intervals of $\pm 1\sigma$ are listed as well. If the confidence interval < 0.005, a value of 0.00 is listed. The power law amplitude *A*, the contrast $\Delta\rho$ and the constant background *B* are given in arbitrary units because the experimental scattering intensities could not be obtained on absolute scale. The power law exponent *D* and the volume fraction *η* are dimensionless quantities.

T/°C	*A*	*D / []*	$\Delta\rho$	r_x */ nm*	r_z */ nm*	r_{eff} */ nm*	*η / []*	*B*
**25**	2.71 ± 0.02	4.14 ± 0.00	2.38 ± 0.08	1.50 ± 0.05	3.2 ± 0.1	1.70 ± 0.03	0.52 ± 0.03	1.9 ± 0.1
**35**	2.76 ± 0.02	4.13 ± 0.00	2.37 ± 0.07	1.50 ± 0.05	3.2 ± 0.1	1.71 ± 0.03	0.53 ± 0.02	1.8 ± 0.1
**45**	3.05 ± 0.03	4.09 ± 0.00	2.4 ± 0.1	1.50 ± 0.07	3.2 ± 0.1	1.73 ± 0.03	0.54 ± 0.02	1.7 ± 0.1
**55**	2.64 ± 0.03	4.14 ± 0.00	2.42 ± 0.09	1.50 ± 0.06	3.2 ± 0.1	1.73 ± 0.03	0.55 ± 0.02	1.6 ± 0.1
**65**	2.47 ± 0.02	4.16 ± 0.00	2.4 ± 0.1	1.50 ± 0.07	3.2 ± 0.1	1.75 ± 0.03	0.56 ± 0.02	1.6 ± 0.1
**75**	2.38 ± 0.02	4.17 ± 0.00	2.40 ± 0.09	1.50 ± 0.06	3.2 ± 0.1	1.75 ± 0.02	0.56 ± 0.02	1.6 ± 0.1
**85**	2.77 ± 0.04	4.12 ± 0.00	2.5 ± 0.2	1.50 ± 0.09	3.1 ± 0.2	1.76 ± 0.03	0.57 ± 0.02	1.4 ± 0.2
**95**	2.56 ± 0.03	4.17 ± 0.01	2.5 ± 0.1	1.50 ± 0.08	2.9 ± 0.2	1.74 ± 0.03	0.55 ± 0.02	1.5 ± 0.2
**105**	1.94 ± 0.03	4.22 ± 0.01	2.61 ± 0.09	1.50 ± 0.06	2.8 ± 0.1	1.73 ± 0.03	0.54 ± 0.02	1.4 ± 0.2
**115**	1.53 ± 0.02	4.26 ± 0.00	2.58 ± 0.08	1.50 ± 0.06	2.9 ± 0.1	1.72 ± 0.02	0.54 ± 0.02	1.5 ± 0.1

**125**	1.34 ± 0.02	4.25 ± 0.00	2.55 ± 0.09	1.50 ± 0.06	3.0 ± 0.1	1.73 ± 0.02	0.54 ± 0.02	1.7 ± 0.1
**135**	0.94 ± 0.01	4.22 ± 0.01	2.63 ± 0.04	1.50 ± 0.03	3.12 ± 0.06	1.65 ± 0.02	0.47 ± 0.01	2.41 ± 0.07
**145**	0.83 ± 0.02	4.03 ± 0.01	3.03 ± 0.07	1.64 ± 0.02	2.8 ± 0.1	1.50 ± 0.01	0.35 ± 0.01	4.28 ± 0.09

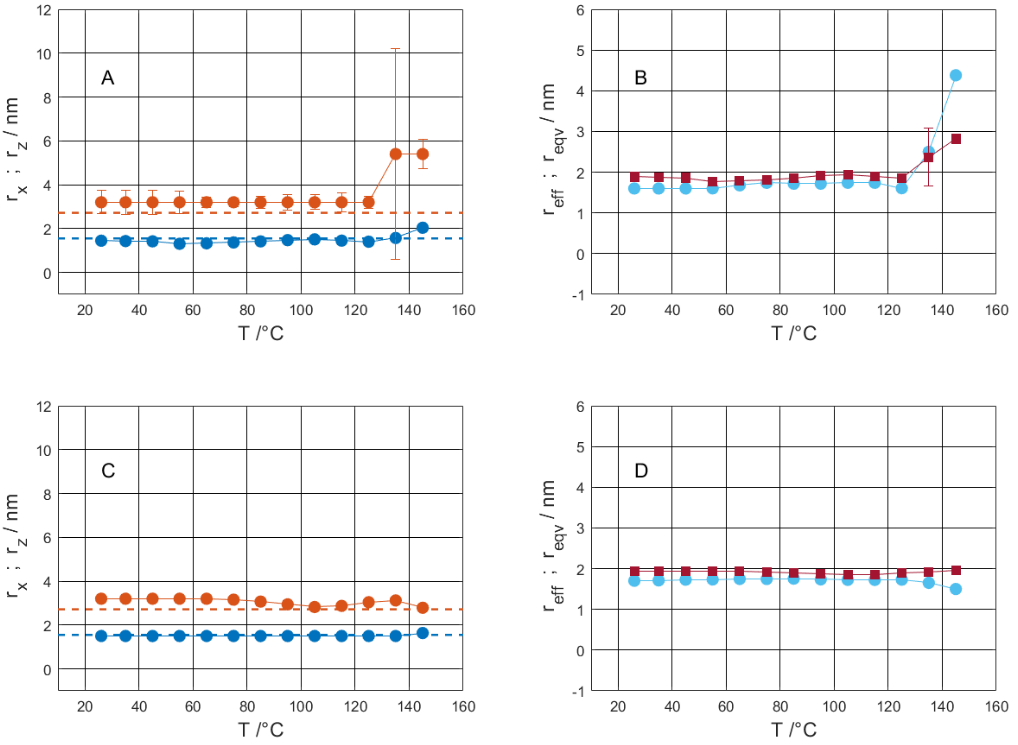

Figure S2: (A,B) Change of radii with increasing temperature for the sample with 20.0 wt-% lysozyme with r_x (●) and r_z (●). The lines indicate the dimensions of the ellipsoid from solution scattering [10]. (C,D) Change of radii with increasing temperature for the sample with 40.0 wt-% lysozyme with r_{eff} (●) and r_{eqv} (●). The equivalent radius r_{eqv} is calculated according to eq. 14.

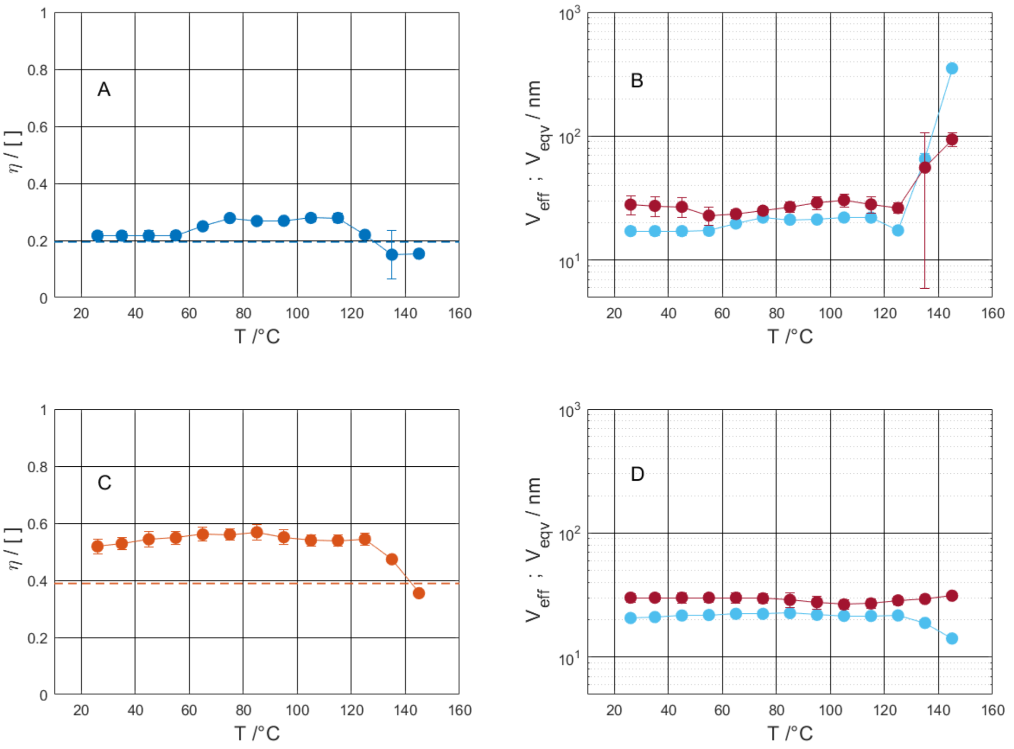

Figure S3: (A,B) Change of volume fraction η and the effective (●) and equivalent (●) volume, with increasing temperature for the sample with 20.0 wt-% lysozyme. (C,D) Change of volume fraction η and the effective (●) and equivalent (●) volume, with increasing temperature for the sample with 40.0 wt-% lysozyme. V_{eff} and V_{eqv} are calculated according to eq. 15 and eq. 8, respectively.
